# Research progress of PYK2 in digestive system diseases

**DOI:** 10.3389/fimmu.2025.1614589

**Published:** 2025-07-17

**Authors:** Yiyao Duan, Mingzhu Xie, Hui Wang, Sijing Chen, Jun Hu, Xujia Chen, Hong Ping Jia, Ningyan Zhang, Ling Peng, Xiang Li, Hameed Ullah Khan, Die Hu, Rong Qin

**Affiliations:** ^1^ Department of Gastroenterology, Yan’ an Hospital Affiliated to Kunming Medical University, Kunming, Yunnan, China; ^2^ Key Laboratory of Tumor Immunological Prevention and Treatment of Yunnan Province, Yan’an Hospital Affiliated to Kunming Medical University, Kunming, Yunnan, China; ^3^ Department of Gynecology, Kunming Maternity and Child Care Hospital, Kunming, Yunnan, China; ^4^ Department of Orthopedics, The First People’s Hospital of Kunming, Kunming, Yunnan, China; ^5^ College of Basic Medical Sciences, Kunming Medical University, Kunming, Yunnan, China; ^6^ Clinical Medical College, Hunan University of Medicine, Huaihua, Hunan, China

**Keywords:** PYK2, digestive system diseases, targeted therapy, tumor progression, signaling pathways

## Abstract

Belonging to the focal adhesion kinase (FAK) family, proline-rich tyrosine kinase 2 (PYK2) is a non-receptor tyrosine kinase, has become a focal point in cancer research owing to its essential participation in the formation and dissemination of tumors. Studies have shown that this kinase controls various cellular activities, including: tumor cell adhesion, growth, multiplication, specialization, and detachment, making it a promising target for developing anticancer drugs. The goal of this review is to analyze the multifaceted role of PYK2 in gastrointestinal disease, focusing on its contribution to tumor progression, associated signaling pathways, and the therapeutic potential of PYK2 inhibitors in improving disease management and prognosis.

## 1 Introduction

Part of the FAK family, PYK2 is referred to as cellular adhesion kinase β (CAK-β), which is mainly distributed in the cytoplasm, and thus belongs to the Cytoplasmic tyrosine kinase (1–3). This enzyme belongs to a unique group of protein kinases that target tyrosine residues on proteins for phosphorylation. Various cell types and tissues universally express PYK2, such as neural tissues, endothelial cells, brain cells, fibroblasts, platelets, and specific hematopoietic cells (4–6).

## 2 Structure and function of PYK2

### 2.1 Structural domains of PYK2

**Figure 1:**
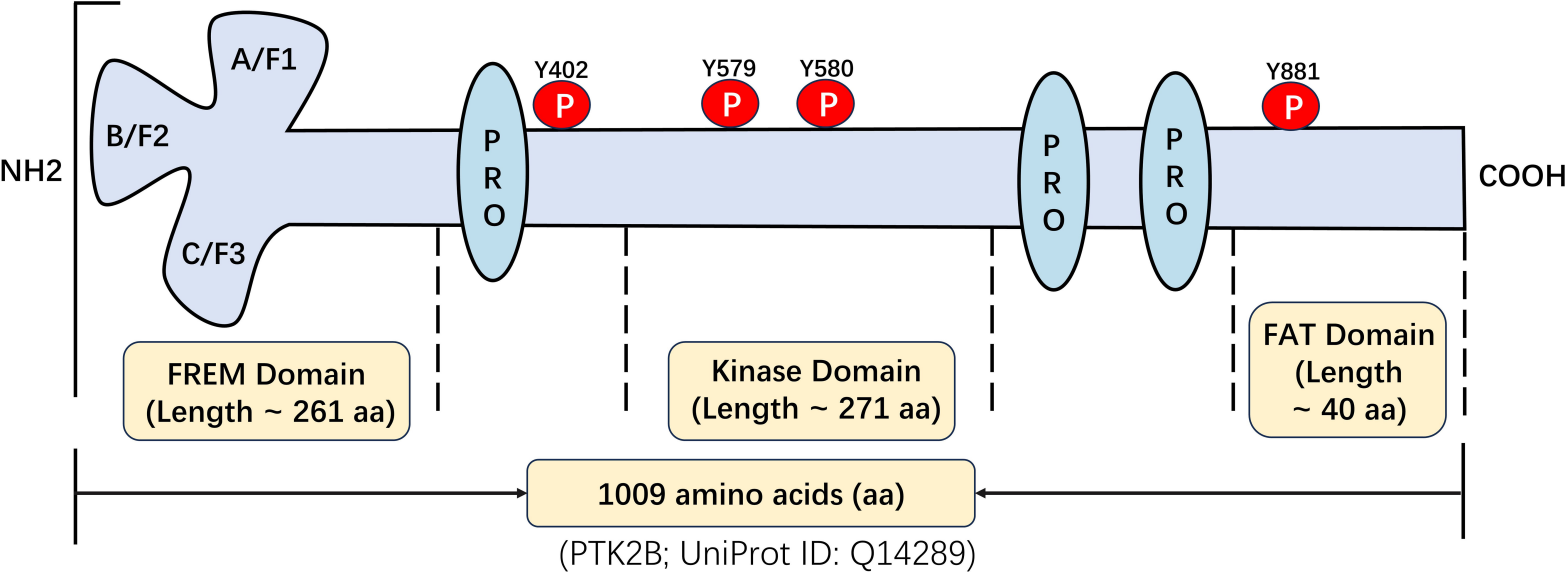
Structural and functional characterization of PYK2: identification of key domains and post-translational modification sites.

PYK2 was initially cloned and identified in 1995 as a gene encoding a protein tyrosine kinase. This gene is found on chromosome 8p21.1 within the human genome, featuring a cDNA sequence that spans 4,048 base pairs and with a molecular mass of ~116 kDa (7). Structural analysis reveals that PYK2 shares significant homology with FAK, exhibiting 46% identity and 65% similarity at the protein level (8). PYK2 contains three core functional domains (9): (1) the FERM (4.1 protein, Ezrin, Radixin, Moesin) domain at the N-terminal: structurally, the FERM domain adopts a compact cloverleaf conformation composed of three distinct structural modules (**Figure 1**). These modules are recognized for their role in facilitating protein-protein and protein-lipid interactions (10); (2) the central domain responsible for tyrosine kinase activity: it contains catalytic residues (Y579/Y580 in the activation loop) and connects to FERM via a conserved linker harboring Y402—a scaffold site for phosphorylation-dependent signaling; and (3) the C-terminal FAT (Focal Adhesion Targeting) domain targets focal adhesions and features the Y881 phosphorylation site, mediates focal adhesion targeting. The spatial organization of these domains determines the function of PYK2 as a signal transduction hub and scaffold.

The primary PYK2 splice variant, PYK2-M, lacks kinase activity due to C-terminal truncation but retains the FERM domain, enabling it to function as a dominant-negative scaffold (11, 12). By competitively binding shared interactors (e.g., integrins, IRF5), PYK2-M disrupts PYK2-H-dependent signaling, suppressing cell migration and pro-inflammatory transcription (13). In digestive pathologies—where PYK2-H drives cancer metastasis (HCC/PDAC) and inflammation (IBD)—PYK2-M may similarly antagonize these processes through kinase-independent scaffolding, though direct evidence remains limited and warrants investigation.

### 2.2 Nuclear functions

#### 2.2.1 Nuclear localization and nucleocytoplasmic shuttling mechanisms of PYK2

The nuclear functions of PYK2 in gastrointestinal tumors are regulated through a multifaceted mechanism involving a classical nuclear localization signal (NLS) located in the F2 subdomain of its FERM domain and a nuclear export signal (NES) embedded within its kinase domain (14). These signals coordinately govern the nucleocytoplasmic shuttling of PYK2. In gastrointestinal tumor cells, calcium influx or mechanical stress activates calcineurin. Activated calcineurin dephosphorylates PYK2 at Ser778, which impairs the function of the NES, leading to enhanced nuclear accumulation of PYK2 (15). For instance, in pancreatic ductal adenocarcinoma (PDAC), increased matrix stiffness induces Piezo1-mediated calcium influx, triggering PYK2 nuclear translocation (16). Furthermore, SUMOylation enhances PYK2 nuclear retention, whereas ubiquitination facilitates its nuclear export. Supporting this, SUMOylation-deficient PYK2 mutants exhibit significantly impaired nuclear accumulation in PDAC models, underscoring the critical role of post-translational modifications in PYK2 nucleocytoplasmic trafficking (17).

#### 2.2.2 Nuclear scaffolding functions of PYK2

As a nuclear scaffolding protein, PYK2 remodels transcriptional networks through multiple mechanisms, including transcription factor cooperativity, chromatin remodeling and epigenetic regulation, and assembly of nuclear signaling complexes, thereby driving the malignant phenotypes of gastrointestinal tumors (18). (1) Transcription Factor Cooperativity: PYK2 directly interacts with GATA-binding protein 4 (GATA4), facilitating its binding to the cyclin D1 promoter and accelerating cell cycle progression (19). In gastric cancer cells, the PYK2-GATA4 complex upregulates c-Myc expression, enhancing tumor cell proliferation.

Additionally, PYK2 interacts with CREB (cAMP response element-binding protein), activating the transcription of inflammatory cytokines such as IL-17A, which contributes to tumor microenvironment remodeling (20, 21). (2) Chromatin Remodeling and Epigenetic Regulation: Notably, in hepatocellular carcinoma models, dissociation of the PYK2-MBD2 complex is associated with activation of Wnt pathway-related genes (e.g., Axin2), promoting tumor cell invasion (22, 23). (3) Assembly of Nuclear Signaling Complexes: PYK2 recruits Src-family kinases (SFKs) in the nucleus, forming a PYK2-Src-Gab1 signaling module that activates the PI3K-AKT pathway (24). In esophageal cancer, this complex enhances the nuclear activity of YAP/TAZ, promoting cancer stem cell maintenance (25). These diverse nuclear scaffolding functions highlight how PYK2, upon nuclear translocation, acts as a central hub for rewiring transcriptional programs to promote gastrointestinal tumorigenesis.

### 2.3 Activation mechanisms of PYK2

Y402 phosphorylation serves as a master switch for PYK2 activation. Diverse stimuli converge on this event through five primary mechanisms:

#### 2.3.1 Cell adhesion-mediated activation

Integrin engagement (e.g., with fibronectin) induces FAK-mediated phosphorylation of Y402. Phosphorylated Y402 recruits Src via its SH2 domain, leading to Src-mediated phosphorylation of Y579/Y580 in the activation loop and full kinase activation (26).

#### 2.3.2 Ca²^+^ and PKC-dependent activation

This mechanism is commonly seen in vascular smooth muscle depolarization or neuronal signal transduction. For example, depolarization of vascular smooth muscle triggers the inflow of Ca2 +, activates PKC, and triggers the above-mentioned core activation pathway (27).

#### 2.3.3 Regulatory role of the FERM domain

Under resting conditions, the FERM domain forms a β-sheet interaction with the linker region of PYK2, occluding the Y402 site. Disruption of this interaction (e.g., via the K60P mutation) exposes Y402, enhancing phosphorylation (28).

#### 2.3.4 Oxidative stress-induced activation

Under oxidative stress (e.g., H_2_O_2_ stimulation), phospholipase D2 (PLD2) activation leads to Y402 phosphorylation, subsequently activating PI3K/Akt pathway to exert a cell-protective effect (29).

**Figure 2:**
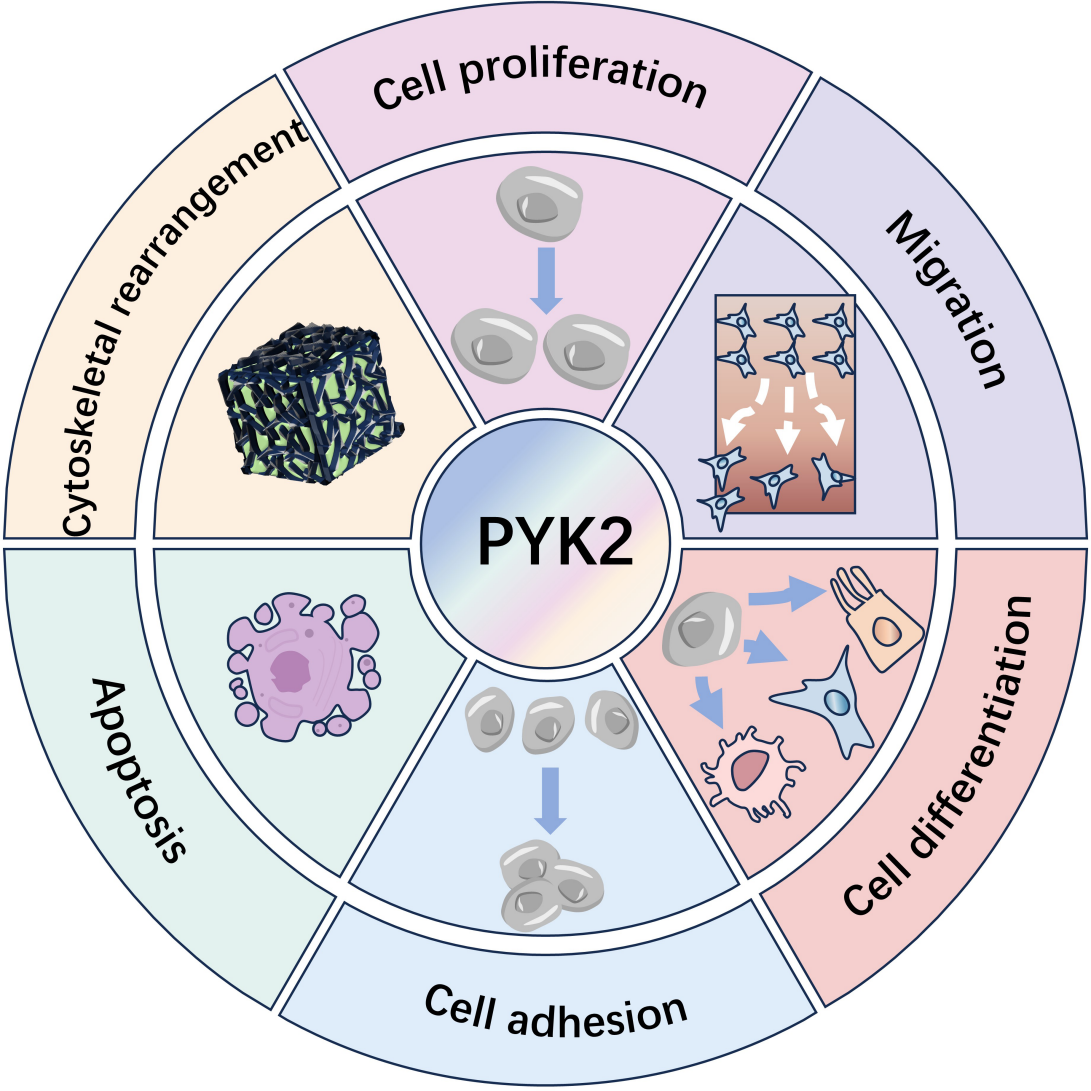
PYK2: A comprehensive overview of its established biological roles.

Through the above-mentioned mechanism, the activated (phosphorylated) PYK2 serves as a critical regulator, coordinating essential cellular activities. These include cytoskeletal remodeling, adhesion signaling, proliferation, motility, phenotypic differentiation, apoptosis modulation, and transcriptional regulation. (**Figure 2**) (7, 30).

### 2.4 Regulatory mechanisms governing PYK2 expression and activity

#### 2.4.1 Post-transcriptional regulation

Regarding the post-transcriptional regulation of PYK2, it is mainly through microRNA-mediated expression regulation. Various microRNAs (miRNAs) inhibit the post-transcriptional expression of PYK2 by binding to the 3’ untranslated region (3’UTR) of PYK2’s mRNA to regulate cancer progression (31–33). For example, in hepatocellular carcinoma (HCC), miR-23b directly targets the 3’UTR of PYK2 and reduces the level of PYK2 protein, thereby inhibiting epithelial-mesenchymal transition (EMT) and tumor metastasis. Mechanistically, miR-23b reduces the expression of matrix metalloproteinases (MMPs) by weakening the PYK2-mediated AKT/mTOR signaling pathway and blocks the invasive ability of cancer cells (31). MiR-214 inhibits PYK2 expression by targeting its mRNA, thereby blocking the PI3K/AKT pathway and inhibiting cell proliferation (32). Down-regulation of miR-517a and miR-517c alleviates their inhibitory effect on PYK2, leading to elevated PYK2 expression. Subsequently, PYK2 promotes cell proliferation by activating ERK1/2 signaling (33).

#### 2.4.2 Post-translational modifications

Post-translational modifications of PYK2 primarily include phosphorylation at Y402, as well as at Y654, which promotes nuclear translocation of β-catenin, which activates the Wnt signaling pathway and drives reprogramming of pancreatic acinar cells and tumor maintenance. This modification relieves ubiquitination degradation of β-catenin and prolongs its half-life (34). In addition to phosphorylation, the activity of PYK2 may also be regulated by ubiquitination (35). Phosphorylated PYK2 may be more readily recognized and ubiquitinated by Cbl-b, forming a “phosphorylation-ubiquitination” cascade that promotes its degradation.

#### 2.4.3 Feedback loops

PYK2 is also involved in the cross-regulation of signaling networks. For example, in PDAC, PYK2 activates the Wnt/β-catenin pathway after phosphorylating β-catenin (Y654), inducing the expression of downstream genes (e.g., c-Myc, Cyclin D1) to promote cell proliferation. Meanwhile, the activation of Wnt signaling can upregulate the expression of PYK2, forming the “PYK2→β-catenin→Wnt→PYK2” feedback, driving the malignant transformation of precancerous cells (34). In addition, in breast cancer, PYK2 activates STAT3 (Y705 site), forming “PYK2→STAT3→PYK2” positive feedback (36). Although this loop was identified in breast cancer, similar mechanisms may operate in digestive tumors given STAT3’s established oncogenic role in PC (37).

These findings collectively demonstrate that PYK2 participates in the cross-regulation of multiple signaling pathways through the formation of positive feedback loops, and thus plays a key role in tumorigenesis and development.

### 2.5 Context-dependent PYK2 function: tissue, microenvironment, epigenetics

Context-dependent function refers to PYK2’s ability to act as either an oncogene or tumor suppressor based on tissue-specific cues, microenvironmental signals, and epigenetic regulation. The paradoxical functions of PYK2 as either an oncogene or tumor suppressor are principally governed by three interconnected determinants: (1) Tissue-specific interacting partners—in GC, PYK2 forms a nuclear complex with p53 and Mdm2 to drive p53 ubiquitination and degradation, thereby disabling a critical tumor-suppressive checkpoint (38–40). Conversely, in HCC, PYK2 physically associates with c-Src to activate MAPK signaling, directly promoting proliferation and invasion (41). (2) Tumor microenvironmental cues—hypoxia in ESCC induces Piezo1-mediated calcium influx, triggering PYK2 phosphorylation at Y402 and subsequent NOX5/c-Abl complex assembly to fuel tumor progression (16, 42); whereas in GC, mechanical stress from peristalsis may modulate RhoA-ROCK signaling through PYK2-p190RhoGEF crosstalk, potentially influencing cell detachment dynamics (43, 44). (3) Epigenetic regulation—miR-23b-mediated PYK2 suppression in HCC inhibits EMT and metastasis (31), whereas downregulation of miR-517a/c in the same malignancy elevates PYK2 expression to activate ERK1/2-driven proliferation (33). This functional plasticity necessitates context-aware therapeutic strategies: PYK2 inhibition may be beneficial in cancers where it acts as an oncogenic scaffold (e.g., ESCC, HCC, PDAC), but could prove detrimental in GC where its tumor-suppressive functions are compromised by downregulation.

## 3 Research progress on the role of PYK2 in digestive system diseases

**Figure 3:**
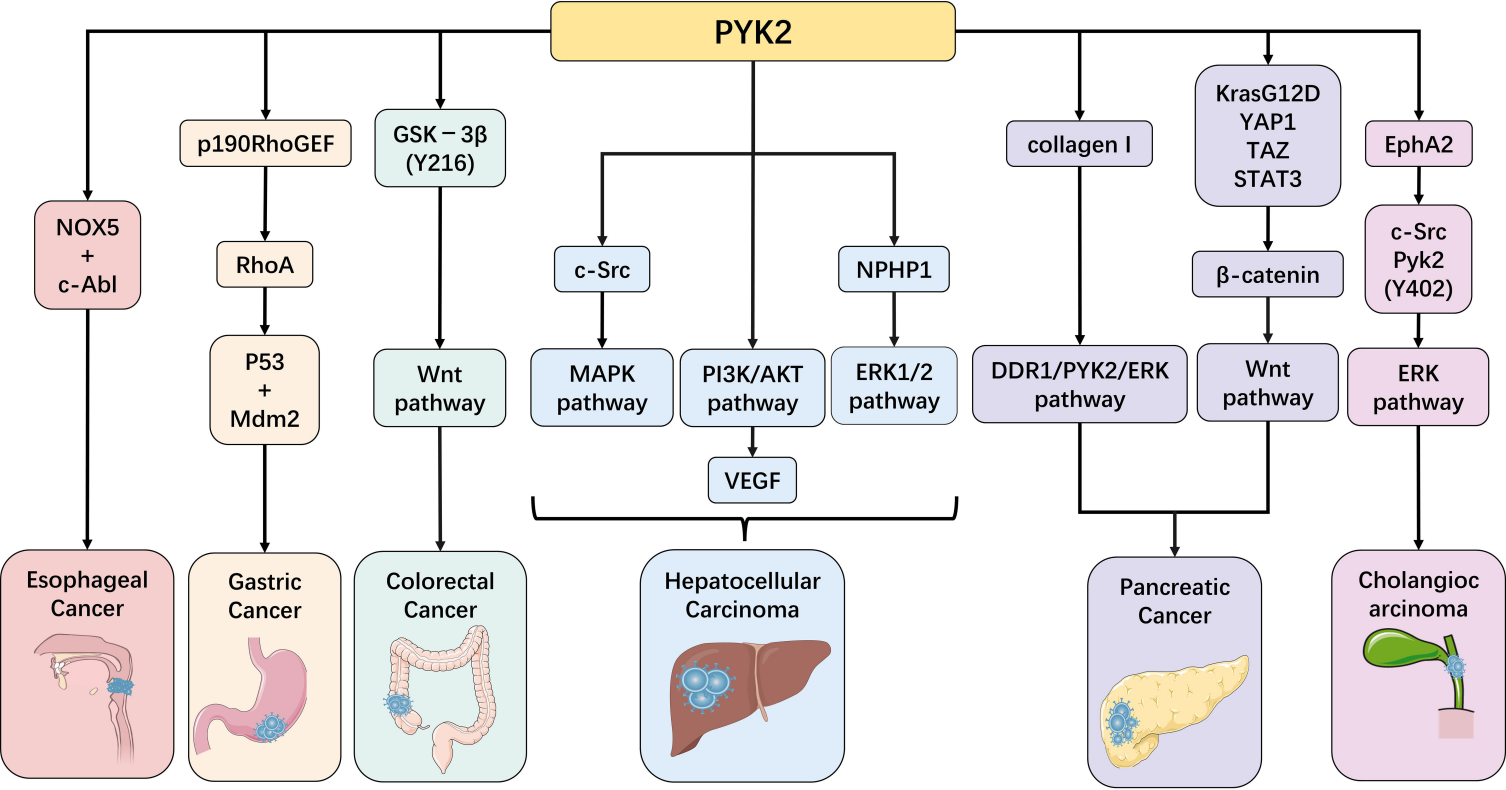
PYK2 in gastrointestinal malignancies: implications in esophageal, gastric, colorectal, hepatic, gallbladder, and pancreatic carcinomas.

**Figure 4:**
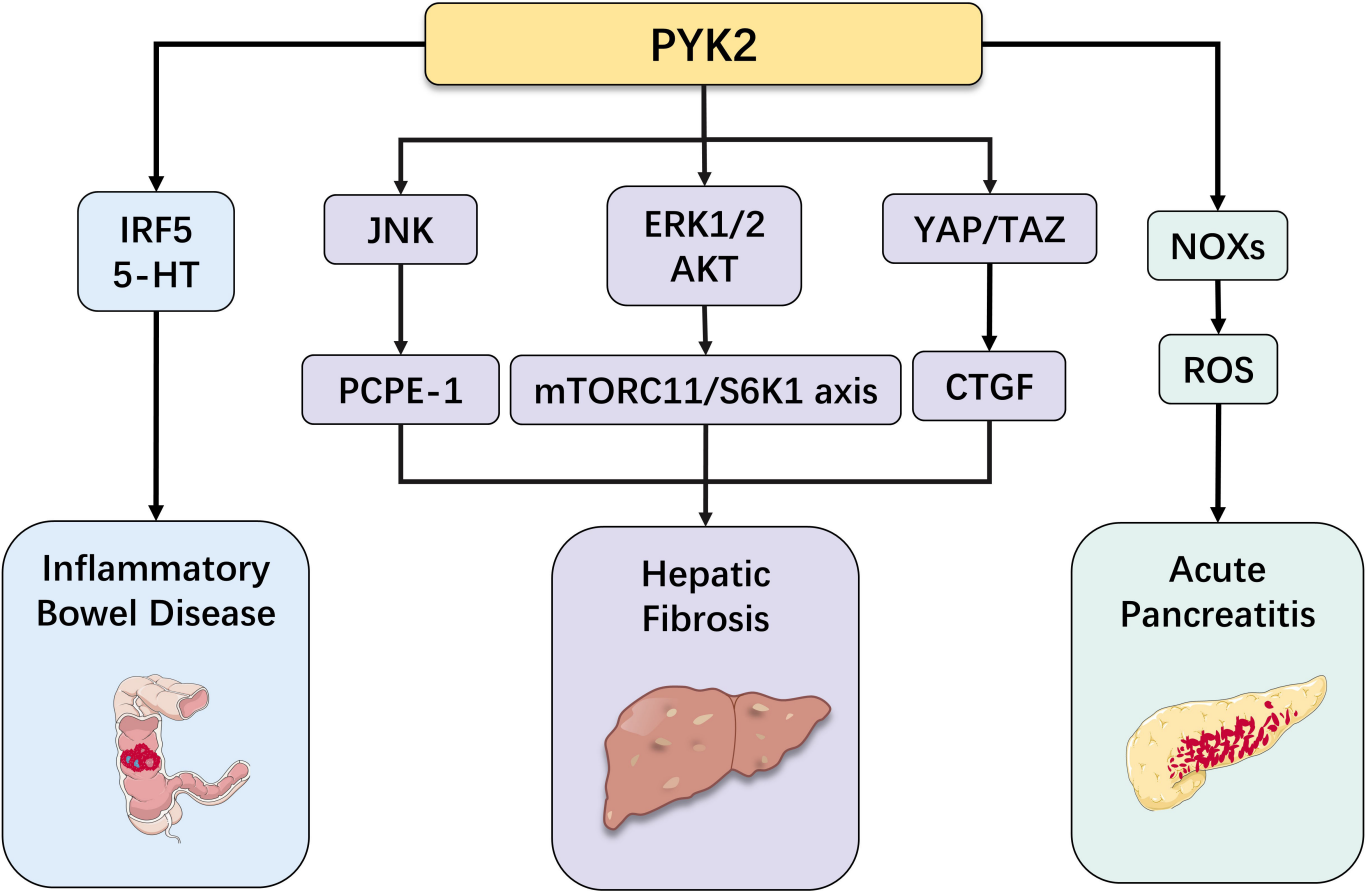
Role of PYK2 in other diseases of the digestive system (inflammatory bowel disease, hepatic fibrosis, acute pancreatitis).

In recent years, with the deepening of PYK2 research, its crucial role in digestive system diseases has become increasingly prominent. Particularly, the regulatory mechanisms of PYK2 in inflammatory diseases and tumorigenesis have emerged as a research hotspot in this field. This review will systematically summarize the latest research progress by focusing on the pivotal functions of PYK2 in various digestive system disorders, including inflammatory bowel disease, hepatic fibrosis, and gastrointestinal tumors (**Figures 3**, **4**). These comprehensive analyses aim to provide novel theoretical foundations and potential intervention strategies for early diagnosis, targeted therapy, and prognosis improvement of related diseases.

### 3.1 The role of PYK2 in digestive system tumors

#### 3.1.1 Esophageal cancer

EC, a common and lethal type of cancer worldwide, constitutes a serious risk to human health. There are two main histological subtypes: esophageal squamous cell carcinoma (ESCC) and esophageal adenocarcinoma (EAC), with ESCC being the most frequent (45). Current therapeutic strategies encompass surgery, radiotherapy, chemotherapy, and endoscopic interventions. Given its highly invasive and metastatic nature, the identification of potential prognostic biomarkers and therapeutic targets is of paramount importance. Current investigations have shown a significant elevation in nicotinamide adenine dinucleotide phosphate oxidase 5 (NOX5) expression levels in ESCC. Mechanistically, hypoxia has been shown to increase intracellular Ca2+ levels, thereby inducing phosphorylation of PYK2 at the Y402 site. This post-translational modification facilitates the interaction between PYK2 and NOX5, leading to the formation of a membrane-associated complex. Furthermore, PYK2 recruits c-Abl, which enhances the activity of NOX5 within this complex, ultimately promoting ESCC progression (42). Although PYK2 does not directly participate in the pathogenesis of EC, it plays a pivotal role in ESCC progression as a scaffold protein that facilitates c-Abl-mediated activation of NOX5 within the PYK2/NOX5 complex. Cisplatin, a commonly used chemotherapeutic agent in adjuvant therapy following radical esophagectomy, has been shown to have its efficacy influenced by PYK2. Moreover, PYK2 expression levels have been correlated with EC prognosis (46). In conclusion, PYK2 critically regulates both tumorigenesis and progression in EC. T Incorporating PYK2 inhibitors into postoperative adjuvant therapy could be a viable strategy for treating EC patients. Additional studies are needed to clarify the exact molecular mechanisms and assess the clinical effectiveness of therapies targeting PYK2.

#### 3.1.2 Gastric carcinoma

Globally, GC represents the fifth most frequently diagnosed malignancy worldwide and ranks among the principal contributors to cancer-associated mortality (47). Elucidating the functional interplay between PYK2 and GC could provide important understanding of the molecular processes involved in disease progression and the creation of new treatments. Notably, studies have demonstrated a marked downregulation of PYK2 expression in GC tissues. Furthermore, clinical evidence has revealed an inverse correlation between the progressive decline in PYK2 expression levels and the advancement of TNM staging, strongly suggesting that function as a tumor suppressor during gastric carcinogenesis (38). The tumor suppressor p53 is a critical guardian of the genome, instrumental in inhibiting GC cell proliferation by inducing cell cycle arrest (primarily at G1/S checkpoint) and apoptosis in response to stress signals (48). Crucially, PYK2 directly antagonizes p53 function, particularly through the formation of a nuclear complex. As detailed in the “Nuclear Functions” section (Section 2.2), the N-terminal FERM domain of PYK2 binds p53 and recruits the E3 ubiquitin ligase Mdm2. This nuclear PYK2-p53-Mdm2 complex facilitates K48-linked polyubiquitination of p53, targeting it for proteasomal degradation (40). The degradation of nuclear p53 by this PYK2-mediated mechanism represents a key pathological event in GC. By reducing functional p53 levels, PYK2 effectively compromises p53-dependent cell cycle arrest, allowing dysregulated proliferation and contributing to tumor progression. This mechanism provides a molecular explanation for the observed inverse correlation between PYK2 expression and GC stage, linking PYK2 downregulation to the loss of a critical tumor suppressor checkpoint. PYK2 may also contributes to tumor metastasis, particularly in the initial detachment phase of cells. Previous studies have established that PYK2 can influence cancer progression by enhancing cell separation, as evidenced by its glucocorticoid-induced upregulation promoting osteoclast detachment (39). In the digestive system, trypsin, a digestive enzyme abundantly present in the stomach, small intestine, and colon, is commonly utilized for cell detachment. Trypsin causes cells to detach by degrading PYK2 via the ubiquitin-lysosome system, but PYK2 can counteract this detachment, though the exact regulatory processes are not yet fully understood (35). Accumulating experimental data indicate that mechanical forces generated by gastric peristalsis could potentially stimulate proliferation in both primary and metastatic GC cell lines, with concomitant modulation of proliferating cell nuclear antigen and p53 expression profiles (43). The tumor suppressor p53 is known to be instrumental in inhibiting the proliferation of GC cells (48). Interestingly, the N-terminal FERM domain of PYK2 can form a complex with p53 and Murine double minute-2 to regulate p53 levels (40). Furthermore, gastrointestinal motility is associated with the RhoA-ROCK signaling pathway, which plays a pivotal role in regulating cancer cell motility and invasion (49). In FAK−/− fibroblasts, PYK2 has been shown to promote RhoA activation and migration through enhanced p190RhoGEF expression (44). Collectively, these findings indicate a tumor-suppressive role for PYK2 in GC. Its downregulation facilitates tumor progression by impairing p53-mediated cell cycle arrest and genomic stability through nuclear complex formation and degradation, and potentially modulating cell detachment and motility pathways. Further investigation into the molecular mechanisms underlying PYK2’s role in GC progression, especially its nuclear scaffolding functions impacting cell cycle regulators beyond p53, may provide novel therapeutic targets for this devastating disease.

#### 3.1.3 Colorectal cancer

CRC, a highly common malignancy globally, is affected by both environmental and genetic factors. Despite groundbreaking developments in both diagnostic technologies and treatment modalities since the turn of the century, there remain unexplored areas that necessitate further investigation. The progression of CRC is a complex, multifactorial process involving PYK2, which contributes not only to colonic smooth muscle contraction but also to intestinal homeostasis, epithelial repair, and tumorigenesis (50, 51). Canonical Wnt signaling constitutes a master regulatory pathway driving colorectal cancer development (52). Mechanistic studies have revealed that elevated FAK/PYK2 levels lead to dephosphorylation of glycogen synthase kinase-3β (GSK-3β) at the Y216 site, facilitating the recruitment of the ubiquitin ligase β-transducin repeats containing proteins (β-TrCP). Ubiquitination of β-catenin through this process activates Wnt signaling (53). These findings suggest that CRC progression is mediated through the PYK2/GSK-3β(Y216)/β-catenin regulatory axis, ultimately influencing cellular metabolism and contributing to malignant transformation. Furthermore, emerging evidence indicates that PYK2 may suppress CRC tumor growth by inhibiting mitochondrial oxidative phosphorylation (OXPHOS) in CRC cells (54). Notably, PYK2 has been identified as a novel independent prognostic marker for colon adenocarcinoma progression following surgical resection (55), with its overexpression demonstrating enhanced cellular proliferation and invasive capabilities. In conclusion, PYK2 critically regulates CRC development and disease outcomes. PYK2’s multifaceted pathway involvement establishes its druggability, warranting further mechanistic and clinical studies for CRC.

#### 3.1.4 Hepatocellular carcinoma

Globally, HCC is the leading type of primary liver cancer. Notably, PYK2 overexpression has been detected in approximately 60% of HCC patients, where it not only promotes cancer cell proliferation but also enhances tumor cell invasiveness (56). Mechanistic investigations have revealed that PYK2 overexpression facilitates its binding with c-Src, forming a PYK2-c-Src signaling complex that undergoes autophosphorylation at Y402. This process activates c-Src, subsequently triggering multiple signaling cascades that activate the MAPK pathway, thereby promoting HCC cell proliferation and invasion (41). Furthermore, TMEM237 overexpression, frequently observed in HCC, has been shown to enhance HCC cell multiplication, movement, invasion, and epithelial-mesenchymal transition (EMT), correlating with poor clinical outcomes. Under hypoxic conditions, the transcriptional activity of the TMEM237 promoter is significantly enhanced through direct binding of HIF-1α (57). The TMEM237-NPHP1 association warrants special attention, as prior research has confirmed NPHP1’s ability to interact with PYK2, resulting in PYK2 phosphorylation and subsequent initiation of the PYK2-dependent ERK1/2 signaling cascade within renal tubular epithelial cells (58). Consequently, when TMEM237 is overexpressed, the interaction between NPHP1 and PYK2 is enhanced, forming a ternary complex that activates the PYK2/ERK1/2 pathway in HCC cells, thereby promoting tumor progression (57). PYK2’s involvement in tumor metastasis has been extensively documented. Researchers have proposed that the activation of the PI3K/AKT pathway by PYK2 enhances VEGF expression in HCC, facilitating peritumoral angiogenesis and inducing tumor metastasis (59). Alternatively, others suggest that PYK2 may enhance metastatic potential by modulating Rac1/RhoA activity to induce EMT (60). independent validation comes from Cao’s work showing miR-23b-mediated PYK2 downregulation effectively curbs EMT-driven HCC invasion (31). Additionally, both miR-214 overexpression and PTK2b/PYK2 knockdown triggered G1-phase arrest, effectively inhibiting malignant cell proliferation through cell cycle disruption (32). Both miR-517a and miR-517c inhibit G2/M phase transition and reduce mitotic activity, at least in part through direct targeting of PYK2, a key regulator of mitotic entry (33). In conclusion, PYK2 is critically involved in HCC progression and metastasis, functioning as both a predictive biomarker for platinum-based chemotherapy resistance and a potential therapeutic target. Further research into PYK2’s molecular mechanisms and its clinical applications may provide novel insights into HCC management strategies.

#### 3.1.5 Pancreatic cancer

Despite notable progress in modern medicine, PC continues to be a highly fatal gastrointestinal cancer, with early detection remaining a significant challenge (61). Pancreatic ductal adenocarcinoma (PDAC) progresses through a multistep mechanism and constitutes the majority of pancreatic cancer cases. This process involves the reprogramming of precancerous acinar cells in response to various stimuli, including Kras oncogene mutations and pancreatitis, leading to pancreatic intraepithelial neoplasia and ultimately progressing to PDAC. During this transformation, alterations in PYK2 and other factors play crucial roles. Mechanistically, Yes-associated protein 1 (YAP) and transcriptional coactivator with PDZ-binding motif (TAZ), effectors of the Hippo pathway, regulate PYK2 transcription via STAT3 mediation. Subsequently, PYK2-mediated phosphorylation of β-catenin drives Wnt pathway activation, establishing PYK2 as a key orchestrator of PDAC tumorigenesis and progression (34, 37, 62). Critically, nuclear-translocated PYK2 acts as a scaffold to potentiate YAP/TAZ transcriptional activity. As detailed in Section 2.2.2, PYK2 recruits Src-family kinases (SFKs) within the nucleus to form a PYK2-Src-YAP/TAZ complex. This complex enhances the nuclear retention and transcriptional output of YAP/TAZ, directly activating genes essential for cancer stem cell maintenance (e.g., SOX9, OCT4) and EMT progression (18, 25). Concurrently, PYK2-phosphorylated β-catenin (pY654) translocates to the nucleus, where it displaces transcriptional repressors (e.g., HDAC1) from TCF/LEF-binding sites, further amplifying Wnt target genes (e.g., c-MYC, CYCD1) (34). This synergistic nuclear crosstalk between PYK2-YAP/TAZ and PYK2-β-catenin axes creates a self-reinforcing transcriptional circuit that drives PDAC aggressiveness. The importance of PYK2 in the development of PDAC is emphasized by these findings. A hallmark of PDAC is its extensive desmoplastic reaction, characterized by substantial type I collagen deposition. This collagen-rich microenvironment signals through integrins and discoidin domain receptor 1 (DDR1), with the DDR1b isoform interacting with PYK2 via Sch1. This interaction induces increased N-cadherin expression and facilitates EMT in PC cells, contributing to PDAC’s tumorigenic properties. During this process, collagen-mediated DDR1 activation induces PYK2-associated signaling pathways, potentially driving collagen-induced tumor progression (63, 64). Furthermore, Recent findings indicate that autophagy levels are crucial in the development and treatment of PDAC (65). Type I collagen activates the DDR1/PYK2/ERK signaling pathway, which primarily mediates autophagosome-lysosome fusion. The use of SH2 super binder to inhibit PDAC cell autophagy through DDR1 regulation has demonstrated antitumor effects (66). In conclusion, PYK2 demonstrates significant associations with pancreatic disease progression. The modulation of PYK2 expression and function holds promise for attenuating or reversing pancreatic pathological processes. Future investigations should prioritize elucidating the molecular mechanisms underlying PYK2’s role in pancreatic pathophysiology and developing targeted therapeutic interventions. These research endeavors may yield significant advancements in the clinical management of pancreatic disorders.

#### 3.1.6 Cholangiocarcinoma

The detection rate of CCA - a biologically aggressive malignancy originating from biliary tract epithelia - has increased significantly with the advent of advanced diagnostic technologies. Despite this progress, research investigating the role of PYK2 in CCA remains limited. A notable study by Cui et al. (67) revealed that in CCA, PYK2 is activated by overexpressed Eph receptor A2 (EphA2). This activation subsequently triggers the PYK2/c-Src signaling pathway, which independently activates the ERK signaling cascade through a Raf/MEK-independent mechanism, ultimately modulating tumor cell invasion and metastatic potential.

### 3.2 The role of Pyk2 in other diseases of the digestive system

#### 3.2.1 Inflammatory bowel disease

IBD, including the two principal phenotypes - ulcerative colitis (UC) and Crohn’s disease (CD), characterized by relapsing-remitting mucosal inflammation (68). Numerous genetic loci play significant roles in IBD pathogenesis, with PYK2 being identified as a nominated pathogenic gene for UC (69). PYK2 not only directly regulates UC progression but also functions as a regulatory factor in its pathological development. Interferon regulatory factor 5 (IRF5), a key immune response regulator, significantly contributes to the development of intestinal inflammation (70). PYK2 exacerbates intestinal inflammation through IRF5 phosphorylation (Tyr-171 in mice/Tyr-172 in humans), a modification that induces pathogenic CD11c+ macrophage accumulation in the inflamed colon (13, 71, 72). Furthermore, studies have demonstrated elevated levels of peripheral serotonin (5-hydroxytryptamine or 5-HT) in patients with intestinal inflammation (73). As an immunomodulatory neuroendocrine peptide, 5-HT not only regulates intestinal homeostasis and tumor biological processes (74, 75), but also induces B cell transformation into regulatory B cells (Bregs), enhancing their capacity to suppress intestinal inflammation (76). PYK2 influences IBD progression through its involvement in 5-HT secretion: upon binding with invariant natural killer T cells, enterochromaffin cells selectively sense lipid antigens, leading to CD1d Tyr332 recruitment and PYK2 activation. Subsequently, PYK2 regulates Kv1.2 channels through tyrosine phosphorylation, triggering Ca2+ influx and subsequent 5-HT release (77).

#### 3.2.2 Hepatic fibrosis

PYK2, a critical regulator of cellular processes, has recently been identified as closely associated with liver health, particularly playing significant roles in lipid metabolism, inflammatory responses, and liver cirrhosis. Its aberrant activity may disrupt lipid homeostasis, exacerbate hepatic inflammation, promote liver fibrosis, and accelerate the progression of cirrhosis. Therefore, in-depth investigation of PYK2’s role in these pathways may provide new hope for developing targeted therapies for liver diseases. Specifically, PYK2 can activate the ERK1/2 and AKT-mediated mTORC1/S6K1 axis, enhancing lipogenesis and leading to hepatic steatosis (78). Furthermore, metabolic dysfunction-associated steatohepatitis is crucial in the development of liver steatosis., characterized by progressive extracellular matrix deposition and sterile hepatic inflammation, potentially culminating in end-stage liver complications including cirrhosis, HCC, and elevated all-cause mortality. When PYK2 is activated, it triggers dimerization associated with its N-terminal FERM domain, resulting in JNK signaling pathway activation. This process increases procollagen C-endopeptidase enhancer 1 in brown adipose tissue, thereby promoting liver fibrosis (79, 80). On the other hand, autophosphorylation of PYK2 at Tyr402 facilitates recognition by Src kinase, forming a PYK2-Src-RhoA ternary complex. This complex leads to YAP/TAZ activation and subsequent induction of connective tissue growth factor, which can also contribute to liver fibrosis (81). In conclusion, PYK2’s complex mechanisms in liver diseases shed light on the pathophysiological processes of hepatic disorders and suggest potential therapeutic targets for developing novel treatment strategies. Further research into PYK2’s molecular interactions and signaling pathways may yield significant advancements in liver disease management.

#### 3.2.3 Acute pancreatitis

AP, a prevalent disorder of the digestive system, is characterized by complex and diverse pathogenesis. Research indicates that inflammatory responses, acinar cell damage, and inappropriate activation of trypsinogen constitute critical biological processes in AP (82). Under normal physiological conditions, pancreatic acinar cells synthesize and secrete digestive enzymes, with trypsinogen and other digestive enzymes remaining in an inactive state. However, when certain pathogenic factors disrupt this balance, leading to inappropriate activation of trypsinogen, acinar cell damage occurs, triggering inflammatory responses. In severe acute pancreatitis (SAP), reactive oxygen species (ROS) and oxidative stress have been closely associated with pancreatic acinar cell injury (83). During this process, PYK2 upregulates the expression of NADPH oxidases (NOXs), promoting excessive ROS generation. As highly reactive molecules, ROS can damage various intracellular biomolecules, including proteins, lipids, and nucleic acids, ultimately compromising cellular function. Furthermore, PYK2 facilitates the activation of AKT and MAPKs, exacerbating cellular oxidative stress and creating a more detrimental cellular environment (84–86). Additionally, PYK2 promotes increased expression of pro-inflammatory cytokines in macrophages (87). The substantial release of these pro-inflammatory cytokines exacerbates inflammatory damage in pancreatic tissue, manifesting as local tissue swelling, pain, and other inflammatory responses, thereby worsening the clinical condition.

## 4 PYK2 in metastasis and genomic alterations

PYK2 orchestrates metastasis in digestive cancers through multifaceted regulation of EMT, cell motility, and tumor microenvironment (TME) remodeling. In HCC, PYK2 promotes EMT by modulating Rac1/RhoA activity, enhancing invasive potential and distant dissemination (60). Concurrently, PYK2-driven PI3K/AKT activation upregulates VEGF, facilitating peritumoral angiogenesis to fuel metastatic spread (59). PDAC studies reveal that nuclear PYK2 scaffolds YAP/TAZ transcriptional complexes, sustaining cancer stemness and EMT via SOX9/OCT4 activation (18, 25). Furthermore, collagen-rich PDAC stroma engages DDR1-PYK2 signaling to induce N-cadherin expression and EMT, linking desmoplasia to metastasis (63, 64). Within the TME, PYK2 phosphorylates IRF5 in macrophages, amplifying pro-inflammatory cytokine release (e.g., IL-17A) and recruiting pathogenic CD11c^+^ macrophages in colitis-associated cancer (13, 71). Genomically, PTK2B (encoding PYK2) exhibits frequent amplifications and missense mutations in gastrointestinal malignancies (e.g., HCC, PDAC), correlating with metastatic progression and therapy resistance. For instance, hypoxia-induced HIF-1α transactivates TMEM237, which stabilizes NPHP1-PYK2 complexes to drive ERK1/2-dependent HCC metastasis (57). PYK2 also confers cisplatin resistance via PI3K/AKT-mediated anti-apoptotic signaling or mitochondrial Lon protein-triggered PYK2-SRC-STAT3 survival pathways (88, 89). Targeting PYK2 with inhibitors (e.g., PF-562,271) disrupts stromal crosstalk and reverses therapy resistance, highlighting its dual role as a metastatic scaffold and genomic driver.

## 5 Therapeutic targeting and overcoming resistance

### 5.1 PYK2 as a therapeutic target

PYK2 has been proven to exert essential functions in gastrointestinal cancers, serving as a signaling hub that integrates inputs from growth factors, integrins, and the tumor microenvironment to drive malignancy. Given its extensive physiological and pathological functions, close association with tumor development, potential roles in other diseases, and the ongoing clinical research on related therapeutic agents, PYK2 indeed represents a promising drug target. This multifaceted involvement positions PYK2 inhibition as a rational strategy for cancer therapy.

### 5.2 Classes of PYK2 inhibitors

Current PYK2-targeted therapeutics fall into three main categories (90): (1) ATP-competitive kinase inhibitors (e.g., PF-562,271, PF-431396), which bind the kinase domain and block phosphorylation (91); (2) allosteric inhibitors (e.g., T6BP), which disrupt scaffolding functions by targeting FERM domain dimerization (79); (3) PROTACs (Proteolysis-Targeting Chimeras), which are experimental agents (e.g., PYK2-PROTAC) that induce ubiquitin-mediated degradation (92). Ongoing clinical trials focus on PF-562,271 in pancreatic cancer (NCT04472174), while PROTACs remain preclinical (93).

### 5.3 Mechanisms of action of key inhibitors

PF-562,271 (Methane sulfonamide diaminopyrimidine), an ATP-competitive inhibitor (94), exhibits potent and reversible inhibition of PYK2 catalytic activity. By blocking PYK2 phosphorylation, it disrupts cancer-associated fibroblast persistence and monocyte recruitment, suppressing tumor growth and metastasis, suppressing tumor growth and metastasis (95). Combining this drug with sunitinib enhances PYK2 inhibition, yielding superior anti-angiogenic and anti-invasive effects (96). PF-431396 (trifluoromethyl pyrimidine) induces apoptosis, inhibits cell cycle progression and metastasis in PDAC and mesothelioma via PYK2 inhibition, and attenuates tumorigenicity in anchorage-independent conditions (97). It also blocks replication of adherent-invasive E. coli(AIEC) in Crohn’s disease and alters Salmonella infection course (98). T6BP blocks PYK2 FERM domain dimerization, attenuating JNK signaling. This reduces hepatic lipid accumulation and cytokine secretion, ameliorating fatty liver disease and fibrosis. T6BP also potentiates CBL-mediated PYK2 ubiquitination and degradation (79).

### 5.4 PYK2-mediated resistance mechanisms

Despite the growing body of research on PYK2 inhibitors, the potential development of acquired drug resistance poses new therapeutic challenges. Studies have shown that PYK2 overexpression increases cisplatin resistance, potentially through PI3K/AKT pathway activation, reduced apoptosis, and upregulation of drug resistance genes (89). Alternatively, cisplatin-induced mitochondrial DNA damage may lead to Lon (a stress protein) overexpression, which, upon binding with the Na+/Ca2+ exchanger, triggers mitochondrial calcium release into the cytoplasm, activating the PYK2-SRC-STAT3 pathway and subsequent BCL-2 expression, ultimately inhibiting apoptosis and contributing to cisplatin resistance (88). Regardless of the dominant mechanism, PYK2 inhibition can enhance tumor necrosis/apoptosis during cisplatin treatment, potentially reducing drug resistance.

### 5.5 Strategies to overcome resistance

Critically, PYK2 functions as a key mediator of acquired resistance to multiple chemotherapeutics, particularly cisplatin, in digestive tumors. Experimental evidence implicates PYK2 overexpression in conferring cisplatin resistance, primarily through two interconnected mechanisms: (1) Activation of the PI3K/AKT survival pathway, suppression of apoptosis, and upregulation of drug resistance genes (89); and/or (2) Cisplatin-induced mitochondrial stress leading to Lon protease overexpression, which triggers calcium release and subsequent activation of the PYK2-SRC-STAT3-BCL-2 anti-apoptotic axis (88). Consequently, targeted inhibition of PYK2 emerges as a rational strategy to overcome or prevent this acquired resistance, potentially sensitizing tumors to cisplatin and improving therapeutic outcomes, as suggested by studies combining PYK2 inhibitors with chemotherapy (95). Furthermore, given its role as a signaling hub and scaffold protein integrating inputs from growth factors, integrins, and the tumor microenvironment, PYK2 may also contribute to resistance against other therapeutic modalities, although this warrants further investigation in digestive cancer contexts. Therefore, targeting PYK2 is highly relevant for overcoming resistance to approved digestive cancer therapies. As a central node in key resistance/survival pathways (e.g., PI3K/AKT, STAT3) (34, 88, 89) and a scaffold for pro-tumorigenic signaling complexes (18, 34), PYK2 inhibition directly disrupts therapy evasion mechanisms. Preclinically, PYK2 inhibitors (e.g., PF-562,271, PF-431396) resensitize tumors to chemotherapeutics like cisplatin by counteracting anti-apoptotic signaling and compensatory survival cascades (95, 96). Furthermore, PYK2’s role in critical tumor microenvironment processes, including stromal crosstalk (e.g., DDR1-PYK2 in PDAC (63), macrophage polarization (e.g., IRF5 phosphorylation 13), and cancer stemness maintenance (e.g., via nuclear YAP/TAZ 18), suggests its contribution to resistance against broader therapies, potentially including targeted agents and immunotherapies. Strategies to mitigate FAK compensation, such as dual FAK/PYK2 inhibitors or exploiting PYK2-specific activation (Ca²^+^/PKC, oxidative stress, nuclear functions) (18, 27, 29, 34, 91, 95), enhance this approach’s feasibility. Thus, PYK2 targeting represents both a direct anti-tumor strategy and a promising combinatorial approach to restore efficacy of approved therapies facing acquired resistance in digestive cancers. While cisplatin’s cytotoxicity involves complex mechanisms beyond these two pathways (99), targeting PYK2 to circumvent drug resistance represents a promising avenue for future research.

Furthermore, given PYK2’s pivotal role as a signaling hub orchestrating multiple oncogenic pathways—including MAPK, PI3K-AKT, and YAP-TAZ—its inhibition presents a compelling rationale for combination therapies with agents targeting these downstream effectors, particularly for overcoming therapy resistance in digestive cancers. Preclinical evidence supports this approach: co-targeting PYK2 and VEGFR (sunitinib) demonstrated superior anti-angiogenic and anti-invasive effects compared to monotherapy in a hepatocellular carcinoma xenograft model, suggesting synergy in disrupting PYK2-driven tumor microenvironment remodeling and survival signaling (96). Specifically, combining PYK2 inhibitors with agents blocking the PI3K-AKT axis could counteract PYK2-mediated anti-apoptotic signaling and drug resistance gene upregulation observed in cisplatin resistance (88, 89). Equally promising is the combination with YAP-TAZ-TEAD pathway inhibitors, as nuclear PYK2 scaffolds transcriptional complexes (e.g., PYK2-Src-YAP/TAZ) critical for cancer stemness maintenance and EMT in PDAC and other GI malignancies (18, 25, 34). Targeting both the upstream activator (PYK2) and the key downstream transcriptional machinery (YAP-TAZ-TEAD) may offer a more comprehensive strategy to dismantle this resilience network and reverse therapy resistance. Future research should prioritize evaluating these rational combinations in models of innate and acquired resistance across diverse digestive tumors. The significant sequence homology (46% identity) and functional redundancy between PYK2 and FAK raise concerns that FAK may compensate for PYK2 inhibition, potentially limiting therapeutic efficacy (7, 8). This compensatory capacity is evidenced by enhanced suppression of tumor progression in HCC models when both kinases are co-inhibited compared to single targeting (41, 95). To circumvent this challenge, strategic approaches focus on dual inhibition or exploiting functional divergence. Dual FAK/PYK2 inhibitors (e.g., PF-562,271 derivatives) simultaneously block compensatory signaling nodes within this kinase family (91, 95). Alternatively, leveraging PYK2-specific regulatory mechanisms—such as its unique activation by Ca²^+^/PKC and oxidative stress (27, 29), and non-redundant nuclear scaffolding functions (e.g., β-catenin Y654 phosphorylation driving Wnt in PDAC; YAP/TAZ complex assembly sustaining cancer stemness) (18, 34) (18, 35)—enables context-selective targeting. This approach minimizes FAK-driven escape while capitalizing on tissue-specific roles of PYK2, such as its tumor-suppressive function in GC versus oncogenic actions in PDAC/HCC (38, 62).

## 6 PYK2 genomic alterations in digestive cancers

Emerging evidence implicates PYK2 (PTK2B) genomic alterations as drivers of digestive carcinogenesis. Analysis of public cohorts (TCGA, cBioPortal) reveals frequent PYK2 amplifications and missense mutations across gastrointestinal malignancies, often correlating with metastatic progression and therapy resistance. The table below summarizes key alterations and their clinical implications (**Table 1**):

**Table 1:** PYK2 genomic alterations in digestive cancers.

Cancer Type	Alteration Frequency	Common Alterations	Clinical Association	References
ESCC	~10-15%	Amplification	Lymph node metastasis	(42, 46)
GC	5-8%	Missense mutations (Y402)	Advanced TNM stage; Tumor suppressor loss	(38)
CRC	12-18%	Amplification	Liver metastasis; Poor post-resection prognosis	(55, 56)
HCC	~20%	Focal gains (8p21.1)	Vascular invasion Recurrence	(57, 60)
PDAC	15-20%	Amplification	Stromal remodeling; Cancer stemness maintenance	(34, 62, 64)
IBD(UC)	GWAS locus	SNP: rs4750316	Disease severity; CD11c^+^ macrophage infiltration	(13, 69)

## 7 Conclusion

Over the past decade, PYK2 has garnered significant attention in the scientific community as a critical regulatory molecule in various cellular processes. In addition to serving as a robust prognostic biomarker for tumor evaluation, this molecule exerts regulatory control over numerous critical signaling cascades that drive the pathogenesis and clinical manifestation of gastrointestinal disorders. This multifaceted role endows PYK2 with unique value in molecular therapy, diagnosis, and prognostic evaluation. Consequently, research on PYK2-targeted drugs has proliferated, particularly in combination with chemotherapeutic agents, demonstrating significant effects in slowing tumor progression, improving prognosis, and even modulating chemotherapy drug resistance. These results highlight the essential role of PYK2 in the onset and progression of diseases affecting the digestive system. However, considering PYK2’s involvement in multiple pathways, several critical questions remain to be addressed. These include potential cross-talk between these pathways, as well as the challenge of balancing antitumor efficacy with the reduction of complications and drug resistance risks in targeted therapy development. Therefore, the widespread application of PYK2-targeted therapy in digestive system diseases remains debatable and requires extensive clinical validation. Furthermore, compared to diseases in other systems, the research landscape between gastrointestinal disorders and PYK2 remains largely unexplored, particularly in specific diseases such as GC and CCA, indicating substantial room for further investigation. In conclusion, while the application prospects of PYK2 in digestive system disease treatment appear promising, it is imperative to maintain a scientifically rigorous approach and continuously promote high-quality clinical research to establish a solid foundation for its future clinical applications. Developing PYK2-targeted therapies requires in-depth research into their molecular mechanisms, possible side effects, and long-term effectiveness to ensure successful and safe clinical implementation.

## References

[1] Shen T Guo Q. Role of pyk2 in human cancers. Med Sci Monitor. (2018) 24:8172–82. doi: 10.12659/msm.913479, PMID: 30425234 PMC6247758

[2] Wang J Bao P Liu Y. Pyk2 regulates sepsis-induced lung injury via ferroptosis. Iran J Basic Med Sci. (2023) 26:1283–90. doi: 10.22038/ijbms.2023.69578.15153, PMID: 37886006 PMC10598808

[3] Zheng J Suo L Zhou Y Jia L Li J Kuang Y. Pyk2 suppresses contextual fear memory in an autophosphorylation-independent manner. J Mol Cell Biol. (2021) 13:808–21. doi: 10.1093/jmcb/mjab057, PMID: 34529077 PMC8782590

[4] Sbrana FV Fiordi B Bordini J Belloni D Barbaglio F Russo L. PYK2 is overexpressed in chronic lymphocytic leukaemia: A potential new therapeutic target. J Cell Mol Med. (2023) 27:576–86. doi: 10.1111/jcmm.17688, PMID: 36747338 PMC9930416

[5] López-Molina L Fernández-Irigoyen J Cifuentes-Díaz C Alberch J Girault J-A Santamaría E. Pyk2 regulates MAMs and mitochondrial dynamics in hippocampal neurons. Cells. (2022) 11:842–60. doi: 10.3390/cells11050842, PMID: 35269464 PMC8909471

[6] Momi S Canino J Vismara M Galgano L Falcinelli E Guglielmini G. Proline-rich tyrosine kinase Pyk2 regulates deep vein thrombosis. Haematologica. (2022) 107:1374–83. doi: 10.3324/haematol.2021.279703, PMID: 35142150 PMC9152972

[7] Zhu X Bao Y Guo Y Yang W. Proline-rich protein tyrosine kinase 2 in inflammation and cancer. Cancers (Basel). (2018) 10:139–52. doi: 10.3390/cancers10050139, PMID: 29738483 PMC5977112

[8] Shen T Guo Q. EGFR signaling pathway occupies an important position in cancer-related downstream signaling pathways of Pyk2. Cell Biol Int. (2020) 44:2–13. doi: 10.1002/cbin.11209, PMID: 31368612 PMC6973235

[9] Palhano Zanela TM Woudenberg A Romero Bello KG Underbakke ES. Activation loop phosphorylation tunes conformational dynamics underlying Pyk2 tyrosine kinase activation. Structure. (2023) 31:447–454.e445. doi: 10.1016/j.str.2023.02.003, PMID: 36870334

[10] Lipinski CA Loftus JC. Targeting Pyk2 for therapeutic intervention. Expert Opin Ther Targets. (2009) 14:95–108. doi: 10.1517/14728220903473194, PMID: 20001213 PMC2943731

[11] Naser R Aldehaiman A Díaz-Galicia E Arold ST. Endogenous control mechanisms of FAK and PYK2 and their relevance to cancer development. Cancers. (2018) 10(6):196–223. doi: 10.3390/cancers10060196, PMID: 29891810 PMC6025627

[12] Meyer AN Gastwirt RF Schlaepfer DD Donoghue DJ. The cytoplasmic tyrosine kinase Pyk2 as a novel effector of fibroblast growth factor receptor 3 activation. J Biol Chem. (2004) 279:28450–7. doi: 10.1074/jbc.M403335200, PMID: 15105428

[13] Ryzhakov G Almuttaqi H Corbin AL Berthold DL Khoyratty T Eames HL. Defactinib inhibits PYK2 phosphorylation of IRF5 and reduces intestinal inflammation. Nat Commun. (2021) 12:6702. doi: 10.1038/s41467-021-27038-5, PMID: 34795257 PMC8602323

[14] Kim SJ Fernandez-Martinez J Nudelman I Shi Y Zhang W Raveh B. Integrative structure and functional anatomy of a nuclear pore complex. Nature. (2018) 555:475–82. doi: 10.1038/nature26003, PMID: 29539637 PMC6022767

[15] Borkúti P Kristó I Szabó A Kovács Z Vilmos P. FERM domain-containing proteins are active components of the cell nucleus. Life Sci Alliance. (2024) 7:1–15. doi: 10.26508/lsa.202302489, PMID: 38296350 PMC10830384

[16] Pan H Zhang X Zhu S Zhu B Wu D Yan J. Piezo1 mediates glycolysis-boosted pancreatic ductal adenocarcinoma chemoresistance within a biomimetic three-dimensional matrix stiffness. ACS Biomater Sci Eng. (2024) 10:7632–46. doi: 10.1021/acsbiomaterials.4c01319, PMID: 39556518

[17] Uzoma I Hu J Cox E Xia S Zhou J Rho HS. Global identification of small ubiquitin-related modifier (SUMO) substrates reveals crosstalk between SUMOylation and phosphorylation promotes cell migration. Mol Cell Proteomics. (2018) 17:871–88. doi: 10.1074/mcp.RA117.000014, PMID: 29438996 PMC5930406

[18] Xie W Yu X Yang Q Ke N Wang P Kong H. An immunomechanical checkpoint PYK2 governs monocyte-to-macrophage differentiation in pancreatic cancer. Cancer Discov. (2025). doi: 10.1158/2159-8290.Cd-24-1712, PMID: 40338055

[19] Jeong K Kim JH Murphy JM Park H Kim SJ Rodriguez YAR. Nuclear focal adhesion kinase controls vascular smooth muscle cell proliferation and neointimal hyperplasia through GATA4-mediated cyclin D1 transcription. Circ Res. (2019) 125:152–66. doi: 10.1161/circresaha.118.314344, PMID: 31096851 PMC6702425

[20] Guan X Liu Y An Y Wang X Wei L Qi X. FAK family kinases: A potential therapeutic target for atherosclerosis. Diabetes Metab Syndr Obes. (2024) 17:3151–61. doi: 10.2147/dmso.S465755, PMID: 39220801 PMC11363942

[21] Miller BA. TRPM2 in cancer. Cell Calcium. (2019) 80:8–17. doi: 10.1016/j.ceca.2019.03.002, PMID: 30925291 PMC6545160

[22] Liu J Feng W Liu M Rao H Li X Teng Y. Stomach-specific c-Myc overexpression drives gastric adenoma in mice through AKT/mammalian target of rapamycin signaling. Bosn J Basic Med Sci. (2021) 21:434–46. doi: 10.17305/bjbms.2020.4978, PMID: 33259779 PMC8292868

[23] Ba M Long H Yan Z Wang S Wu Y Tu Y. BRD4 promotes gastric cancer progression through the transcriptional and epigenetic regulation of c-MYC. J Cell Biochem. (2018) 119:973–82. doi: 10.1002/jcb.26264, PMID: 28681984

[24] Zhang W Yu L Xu C Tang T Cao J Chen L. PLEK2 activates the PI3K/AKT signaling pathway to drive lung adenocarcinoma progression by upregulating SPC25. Cell Biol Int. (2024) 48:1285–300. doi: 10.1002/cbin.12197, PMID: 38894536

[25] Muramatsu T Imoto I Matsui T Kozaki K Haruki S Sudol M. YAP is a candidate oncogene for esophageal squamous cell carcinoma. Carcinogenesis. (2011) 32:389–98. doi: 10.1093/carcin/bgq254, PMID: 21112960

[26] Zhao M Finlay D Zharkikh I Vuori K. Novel role of src in priming pyk2 phosphorylation. PloS One. (2016) 11:e0149231. doi: 10.1371/journal.pone.0149231, PMID: 26866924 PMC4750869

[27] Mills RD Mita M Nakagawa J Shoji M Sutherland C Walsh MP. A role for the tyrosine kinase Pyk2 in depolarization-induced contraction of vascular smooth muscle. J Biol Chem. (2015) 290:8677–92. doi: 10.1074/jbc.M114.633107, PMID: 25713079 PMC4423659

[28] Zanela TMP Zangiabadi M Zhao Y Underbakke ES. Molecularly imprinted nanoparticles reveal regulatory scaffolding features in Pyk2 tyrosine kinase. RSC Chem Biol. (2024) 5:447–53. doi: 10.1039/d3cb00228d, PMID: 38725907 PMC11078204

[29] Banno Y Ohguchi K Matsumoto N Koda M Ueda M Hara A. Implication of phospholipase D2 in oxidant-induced phosphoinositide 3-kinase signaling via Pyk2 activation in PC12 cells. J Biol Chem. (2005) 280:16319–24. doi: 10.1074/jbc.M410903200, PMID: 15705590

[30] Lee D Hong J-H. Activated pyK2 and its associated molecules transduce cellular signaling from the cancerous milieu for cancer metastasis. Int J Mol Sci. (2022) 23:15475–88. doi: 10.3390/ijms232415475, PMID: 36555115 PMC9779422

[31] Cao J Liu J Long J Fu J Huang L Li J. microRNA-23b suppresses epithelial-mesenchymal transition (EMT) and metastasis in hepatocellular carcinoma via targeting Pyk2. BioMed Pharmacother. (2017) 89:642–50. doi: 10.1016/j.biopha.2017.02.030, PMID: 28262617

[32] Chen C Tao Z Li Y Li J Xu Y. MicroRNA214 expression inhibits HCC cell proliferation through PTK2b/ Pyk2. Cell Mol Biol (Noisy-le-grand). (2022) 68:20–5. doi: 10.14715/cmb/2022.68.1.4, PMID: 35809332

[33] Liu RF Xu X Huang J Fei QL Chen F Li YD. Down-regulation of miR-517a and miR-517c promotes proliferation of hepatocellular carcinoma cells via targeting Pyk2. Cancer Lett. (2013) 329:164–73. doi: 10.1016/j.canlet.2012.10.027, PMID: 23142219

[34] Gao C Chen G Zhang DH Zhang J Kuan S-F Hu W. PYK2 is involved in premalignant acinar cell reprogramming and pancreatic ductal adenocarcinoma maintenance by phosphorylating β-cateninY654. Cell Mol Gastroenterol Hepatol. (2019) 8:561–78. doi: 10.1016/j.jcmgh.2019.07.004, PMID: 31330317 PMC6889497

[35] Fan Y Qu X Ma Y Qu J Liu Y Hu X. Cbl-b accelerates trypsin-induced cell detachment through ubiquitination and degradation of proline-rich tyrosine kinase 2. Tumor Biol. (2014) 35:11129–35. doi: 10.1007/s13277-014-2296-z, PMID: 25099615

[36] Verma N Keinan O Selitrennik M Karn T Filipits M Lev S. PYK2 sustains endosomal-derived receptor signalling and enhances epithelial-to-mesenchymal transition. Nat Commun. (2015) 6:6064. doi: 10.1038/ncomms7064, PMID: 25648557

[37] Gruber R Panayiotou R Nye E Spencer-Dene B Stamp G Behrens A. YAP1 and TAZ control pancreatic cancer initiation in mice by direct up-regulation of JAK–STAT3 signaling. Gastroenterology. (2016) 151:526–39. doi: 10.1053/j.gastro.2016.05.006, PMID: 27215660 PMC5007286

[38] Guo HJ Wang X Liu YC Wan YL Yin HF Li T. Expression of proline-rich tyrosine kinase-2 (Pyk2) in gastric carcinoma and its significance. Beijing Da Xue Xue Bao Yi Xue Ban. (2005) 37:261–4., PMID: 15968315

[39] Sato AY Cregor M McAndrews K Li T Condon KW Plotkin LI. Glucocorticoid-induced bone fragility is prevented in female mice by blocking pyk2/anoikis signaling. Endocrinology. (2019) 160:1659–73. doi: 10.1210/en.2019-00237, PMID: 31081900 PMC6591015

[40] Lim S-T Miller NLG Nam J-O Chen XL Lim Y Schlaepfer DD. Pyk2 inhibition of p53 as an adaptive and intrinsic mechanism facilitating cell proliferation and survival. J Biol Chem. (2010) 285:1743–53. doi: 10.1074/jbc.M109.064212, PMID: 19880522 PMC2804332

[41] Sun CK Man K Ng KT Ho JW Lim ZX Cheng Q. Proline-rich tyrosine kinase 2 (Pyk2) promotes proliferation and invasiveness of hepatocellular carcinoma cells through c-Src/ERK activation. Carcinogenesis. (2008) 29:2096–105. doi: 10.1093/carcin/bgn203, PMID: 18765415

[42] Chen J Wang Y Zhang W Zhao D Zhang L Fan J. Membranous NOX5-derived ROS oxidizes and activates local Src to promote Malignancy of tumor cells. Signal Transduction Targeted Ther. (2020) 5:139–50. doi: 10.1038/s41392-020-0193-z, PMID: 32792487 PMC7426961

[43] Zhao J Wang R Zhang J Zhao Y Qiao S Crouzier T. A novel 4D cell culture mimicking stomach peristalsis altered gastric cancer spheroids growth and Malignance. Biofabrication. (2021) 13:035028. doi: 10.1088/1758-5090/abf6bf, PMID: 33836517

[44] Lim Y Lim S-T Tomar A Gardel M Bernard-Trifilo JA Chen XL. PyK2 and FAK connections to p190Rho guanine nucleotide exchange factor regulate RhoA activity, focal adhesion formation, and cell motility. J Cell Biol. (2008) 180:187–203. doi: 10.1083/jcb.200708194, PMID: 18195107 PMC2213606

[45] Zhu H Ma X Ye T Wang H Wang Z Liu Q. Esophageal cancer in China: Practice and research in the new era. Int J Cancer. (2023) 152:1741–51. doi: 10.1002/ijc.34301, PMID: 36151861

[46] Zhu T Yang Q Shao J Chen Z Cai B Mao G. Pyk2 level is a novel prognostic marker for patients with esophageal squamous cell carcinoma after radical surgery. Virchows Archiv. (2021) 479:905–17. doi: 10.1007/s00428-021-03153-y, PMID: 34313839

[47] López MJ Carbajal J Alfaro AL Saravia LG Zanabria D Araujo JM. Characteristics of gastric cancer around the world. Crit Rev Oncol Hematol. (2023) 181:103841. doi: 10.1016/j.critrevonc.2022.103841, PMID: 36240980

[48] Ji W Ma J Zhang H Zhong HUA Li LEI Ding NA. Role of p53β in the inhibition of proliferation of gastric cancer cells expressing wild-type or mutated p53. Mol Med Rep. (2015) 12:691–5. doi: 10.3892/mmr.2015.3370, PMID: 25695150

[49] Matsuoka T. Rho/ROCK signaling in motility and metastasis of gastric cancer. World J Gastroenterol. (2014) 20(38):13756–66. doi: 10.3748/wjg.v20.i38.13756, PMID: 25320513 PMC4194559

[50] Tong L Ao J-P Lu H-L Huang X Zang J-Y Liu S-H. Tyrosine kinase pyk2 is involved in colonic smooth muscle contraction via the rhoA/ROCK pathway. Physiol Res. (2018) 68:89–98. doi: 10.33549/physiolres.933857, PMID: 30433799

[51] Thomas KS Owen KA Conger K Llewellyn RA Bouton AH Casanova JE. Non-redundant functions of FAK and Pyk2 in intestinal epithelial repair. Sci Rep. (2019) 9:4497. doi: 10.1038/s41598-019-41116-1, PMID: 30872746 PMC6418130

[52] Nie X Liu H Liu L Wang Y-D Chen W-D. Emerging roles of wnt ligands in human colorectal cancer. Front Oncol. (2020) 10:1341. doi: 10.3389/fonc.2020.01341, PMID: 32923386 PMC7456893

[53] Gao C Chen G Kuan SF Zhang DH Schlaepfer DD Hu J. FAK/PYK2 promotes the Wnt/β-catenin pathway and intestinal tumorigenesis by phosphorylating GSK3β. Elife. (2015) 4:e10072.001–e10072.017. doi: 10.7554/eLife.10072, PMID: 26274564 PMC4558782

[54] Qin R Huang Y Yao Y Wang L Zhang Z Huang W. The role and molecular mechanism of metabolic reprogramming of colorectal cancer by UBR5 through PYK2 regulation of OXPHOS expression study. J Biochem Mol Toxicol. (2023) 37:e23376. doi: 10.1002/jbt.23376, PMID: 37098808

[55] Liu S Chen L Xu Y. Significance of PYK2 level as a prognosis predictor in patients with colon adenocarcinoma after surgical resection. OncoTargets Ther. (2018) 11:7625–34. doi: 10.2147/ott.S169531, PMID: 30464511 PMC6217216

[56] Sun CK Ng KT Sun BS Ho JWY Lee TK Ng I. The significance of proline-rich tyrosine kinase2 (Pyk2) on hepatocellular carcinoma progression and recurrence. Br J Cancer. (2007) 97:50–7. doi: 10.1038/sj.bjc.6603827, PMID: 17551499 PMC2359657

[57] Chen T Wang L Chen C Li R Zhu N Liu R. HIF-1α-activated TMEM237 promotes hepatocellular carcinoma progression via the NPHP1/Pyk2/ERK pathway. Cell Mol Life Sci. (2023) 80:120–34. doi: 10.1007/s00018-023-04767-y, PMID: 37041420 PMC11072547

[58] Benzing T Gerke P Höpker K Hildebrandt F Kim E Walz G. Nephrocystin interacts with Pyk2, p130(Cas), and tensin and triggers phosphorylation of Pyk2. Proc Natl Acad Sci U.S.A. (2001) 98:9784–9. doi: 10.1073/pnas.171269898, PMID: 11493697 PMC55530

[59] Cao J Chen Y Fu J Qian Y-W Ren Y-B Su B. High expression of proline-rich tyrosine kinase2 is associated with poor survival of hepatocellular carcinoma via regulating phosphatidylinositol 3-kinase/AKT pathway. Ann Surg Oncol. (2012) 20:312–23. doi: 10.1245/s10434-012-2372-9, PMID: 22618716

[60] Dent P Sun CK Ng KT Lim ZX Cheng Q Lo CM. Proline-rich tyrosine kinase 2 (Pyk2) promotes cell motility of hepatocellular carcinoma through induction of epithelial to mesenchymal transition. PloS One. (2011) 6(4):e18878. doi: 10.1371/journal.pone.0018878, PMID: 21533080 PMC3080371

[61] Hu J-X Zhao C-F Chen W-B Liu Q-C Li Q-W Lin Y-Y. Pancreatic cancer: A review of epidemiology, trend, and risk factors. World J Gastroenterol. (2021) 27:4298–321. doi: 10.3748/wjg.v27.i27.4298, PMID: 34366606 PMC8316912

[62] Means AL. PYK2 at the intersection of signaling pathways in pancreatic cancer. Cell Mol Gastroenterol Hepatol. (2019) 8:651–2. doi: 10.1016/j.jcmgh.2019.08.007, PMID: 31525324 PMC6889775

[63] Huang H Svoboda RA Lazenby AJ Saowapa J Chaika N Ding K. Up-regulation of N-cadherin by collagen I-activated discoidin domain receptor 1 in pancreatic cancer requires the adaptor molecule shc1. J Biol Chem. (2016) 291:23208–23. doi: 10.1074/jbc.M116.740605, PMID: 27605668 PMC5087738

[64] Aguilera KY Huang H Du W Hagopian MM Wang Z Hinz S. Inhibition of discoidin domain receptor 1 reduces collagen-mediated tumorigenicity in pancreatic ductal adenocarcinoma. Mol Cancer Ther. (2017) 16:2473–85. doi: 10.1158/1535-7163.Mct-16-0834, PMID: 28864681 PMC5669827

[65] Li J Chen X Kang R Zeh H Klionsky DJ Tang D. Regulation and function of autophagy in pancreatic cancer. Autophagy. (2020) 17:3275–96. doi: 10.1080/15548627.2020.1847462, PMID: 33161807 PMC8632104

[66] Xu H Tan M Hou G-Q Sang Y-Z Lin L Gan X-C. Blockade of DDR1/PYK2/ERK signaling suggesting SH2 superbinder as a novel autophagy inhibitor for pancreatic cancer. Cell Death Dis. (2023) 14:811. doi: 10.1038/s41419-023-06344-4, PMID: 38071340 PMC10710504

[67] Cui XD Lee MJ Kim JH Hao PP Liu L Yu GR. Activation of mammalian target of rapamycin complex 1 (mTORC1) and Raf/Pyk2 by growth factor-mediated Eph receptor 2 (EphA2) is required for cholangiocarcinoma growth and metastasis. Hepatology. (2013) 57:2248–60. doi: 10.1002/hep.26253, PMID: 23315987

[68] Ning S Zhang Z Zhou C Wang B Liu Z Feng B. Cross-talk between macrophages and gut microbiota in inflammatory bowel disease: a dynamic interplay influencing pathogenesis and therapy. Front Med. (2024) 11:1457218. doi: 10.3389/fmed.2024.1457218, PMID: 39355844 PMC11443506

[69] Liu JZ van Sommeren S Huang H Ng SC Alberts R Takahashi A. Association analyses identify 38 susceptibility loci for inflammatory bowel disease and highlight shared genetic risk across populations. Nat Genet. (2015) 47:979–86. doi: 10.1038/ng.3359, PMID: 26192919 PMC4881818

[70] Liang C Tang Y Gao X Lei N Luo Y Chen P. Depression exacerbates dextran sulfate sodium-induced colitis via IRF5-mediated macrophage polarization. Digestive Dis Sci. (2022) 68:1269–79. doi: 10.1007/s10620-022-07679-2, PMID: 36088512

[71] Corbin AL Gomez-Vazquez M Berthold DL Attar M Arnold IC Powrie FM. IRF5 guides monocytes toward an inflammatory CD11c(+) macrophage phenotype and promotes intestinal inflammation. Sci Immunol. (2020) 5:eabc708. doi: 10.1126/sciimmunol.aax6085, PMID: 32444476 PMC7611075

[72] Hegarty LM Jones GR Bain CC. Macrophages in intestinal homeostasis and inflammatory bowel disease. Nat Rev Gastroenterol Hepatol. (2023) 20:538–53. doi: 10.1038/s41575-023-00769-0, PMID: 37069320

[73] Jørandli JW Thorsvik S Skovdahl HK Kornfeld B Sæterstad S Gustafsson BI. The serotonin reuptake transporter is reduced in the epithelium of active Crohn's disease and ulcerative colitis. Am J Physiol Gastrointest Liver Physiol. (2020) 319:G761–g768. doi: 10.1152/ajpgi.00244.2020, PMID: 32967429

[74] Ye D Xu H Tang Q Xia H Zhang C Bi F. The role of 5-HT metabolism in cancer. Biochim Biophys Acta (BBA) - Rev Cancer. (2021) 1876:1–20. doi: 10.1016/j.bbcan.2021.188618, PMID: 34428515

[75] Chen Z Luo J Li J Kim G Stewart A Urban JF. Interleukin-33 promotes serotonin release from enterochromaffin cells for intestinal homeostasis. Immunity. (2021) 54:151–163.e156. doi: 10.1016/j.immuni.2020.10.014, PMID: 33220232 PMC7856083

[76] Wan M Ma Z Han J Rao M Hu F Gao P. 5-HT induces regulatory B cells in fighting against inflammation-driven ulcerative colitis. Int Immunopharmacol. (2023) 125:111042. doi: 10.1016/j.intimp.2023.111042, PMID: 37866311

[77] Luo J Chen Z Castellano D Bao B Han W Li J. Lipids regulate peripheral serotonin release via gut CD1d. Immunity. (2023) 56:1533–1547.e1537. doi: 10.1016/j.immuni.2023.06.001, PMID: 37354904 PMC10527042

[78] Ashraf S Ashraf N Yilmaz G Harmancey R. Crosstalk between beta-adrenergic and insulin signaling mediates mechanistic target of rapamycin hyperactivation in liver of high-fat diet-fed male mice. Physiol Rep. (2021) 9:e14958. doi: 10.14814/phy2.14958, PMID: 34231324 PMC8261682

[79] Xu M Zhao J Zhu L Ge C Sun Y Wang R. Targeting PYK2 with heterobifunctional T6BP helps mitigate MASLD and MASH-HCC progression. J Hepatol. (2024) 1–24. doi: 10.1016/j.jhep.2024.08.029, PMID: 39260704

[80] Hsiao YT Yoshida Y Okuda S Abe M Mizuno S Takahashi S. PCPE-1, a brown adipose tissue-derived cytokine, promotes obesity-induced liver fibrosis. EMBO J. (2024) 43:4846–69. doi: 10.1038/s44318-024-00196-0, PMID: 39160276 PMC11535236

[81] Kim J Kang W Kang SH Park SH Kim JY Yang S. Proline-rich tyrosine kinase 2 mediates transforming growth factor-beta-induced hepatic stellate cell activation and liver fibrosis. Sci Rep. (2020) 10:21018. doi: 10.1038/s41598-020-78056-0, PMID: 33273492 PMC7713048

[82] Zerem E Kurtcehajic A Kunosić S Zerem Malkočević D Zerem O. Current trends in acute pancreatitis: Diagnostic and therapeutic challenges. World J Gastroenterol. (2023) 29:2747–63. doi: 10.3748/wjg.v29.i18.2747, PMID: 37274068 PMC10237108

[83] Kong L Deng J Zhou X Cai B Zhang B Chen X. Sitagliptin activates the p62–Keap1–Nrf2 signalling pathway to alleviate oxidative stress and excessive autophagy in severe acute pancreatitis-related acute lung injury. Cell Death Dis. (2021) 12:928–38. doi: 10.1038/s41419-021-04227-0, PMID: 34635643 PMC8505515

[84] Zhang X Yang Y Jing L Zhai W Zhang H Ma Q. Pyruvate kinase M2 contributes to TLR-mediated inflammation and autoimmunity by promoting pyk2 activation. Front Immunol. (2021) 12:680068. doi: 10.3389/fimmu.2021.680068, PMID: 34025679 PMC8138060

[85] Konno T Kohno T Miyakawa M Tanaka H Kojima T. Pyk2 inhibitor prevents epithelial hyperpermeability induced by HMGB1 and inflammatory cytokines in Caco-2 cells. Tissue Barriers. (2021) 9:1890526. doi: 10.1080/21688370.2021.1890526, PMID: 33660567 PMC8078543

[86] Cai Y Yu R Zhang Z Li D Yi B Feng Z. Mettl3/Ythdf2 regulate macrophage inflammation and ROS generation by controlling Pyk2 mRNA stability. Immunol Lett. (2023) 264:64–73. doi: 10.1016/j.imlet.2023.11.004, PMID: 37952687

[87] Murphy JM Jeong K Rodriguez YAR Kim J-H Ahn E-YE Lim S-TS. FAK and Pyk2 activity promote TNF-α and IL-1β-mediated pro-inflammatory gene expression and vascular inflammation. Sci Rep. (2019) 9:7617. doi: 10.1038/s41598-019-44098-2, PMID: 31110200 PMC6527705

[88] Tangeda V Lo YK Babuharisankar AP Chou HY Kuo CL Kao YH. Lon upregulation contributes to cisplatin resistance by triggering NCLX-mediated mitochondrial Ca(2+) release in cancer cells. Cell Death Dis. (2022) 13:241. doi: 10.1038/s41419-022-04668-1, PMID: 35296653 PMC8927349

[89] Maki CG Geng W Ng KTP Sun CKW Yau WL Liu XB. The role of proline rich tyrosine kinase 2 (Pyk2) on cisplatin resistance in hepatocellular carcinoma. PloS One. (2011) 6(11):e27362. doi: 10.1371/journal.pone.0027362, PMID: 22096562 PMC3212555

[90] Yu F Cai M Shao L Zhang J. Targeting protein kinases degradation by PROTACs. Front Chem. (2021) 9:679120. doi: 10.3389/fchem.2021.679120, PMID: 34277564 PMC8279777

[91] Sulzmaier FJ Jean C Schlaepfer DD. FAK in cancer: mechanistic findings and clinical applications. Nat Rev Cancer. (2014) 14:598–610. doi: 10.1038/nrc3792, PMID: 25098269 PMC4365862

[92] Song Y Dong QQ Ni YK Xu XL Chen CX Chen W. Nano-proteolysis targeting chimeras (Nano-PROTACs) in cancer therapy. Int J Nanomedicine. (2024) 19:5739–61. doi: 10.2147/ijn.S448684, PMID: 38882545 PMC11180470

[93] Dawson JC Serrels A Stupack DG Schlaepfer DD Frame MC. Targeting FAK in anticancer combination therapies. Nat Rev Cancer. (2021) 21:313–24. doi: 10.1038/s41568-021-00340-6, PMID: 33731845 PMC8276817

[94] Roberts WG Ung E Whalen P Cooper B Hulford C Autry C. Antitumor activity and pharmacology of a selective focal adhesion kinase inhibitor, PF-562,271. Cancer Res. (2008) 68:1935–44. doi: 10.1158/0008-5472.Can-07-5155, PMID: 18339875

[95] Stokes JB Adair SJ Slack-Davis JK Walters DM Tilghman RW Hershey ED. Inhibition of focal adhesion kinase by PF-562,271 inhibits the growth and metastasis of pancreatic cancer concomitant with altering the tumor microenvironment. Mol Cancer Ther. (2011) 10:2135–45. doi: 10.1158/1535-7163.Mct-11-0261, PMID: 21903606 PMC3213273

[96] Bagi CM Christensen J Cohen DP Roberts WG Wilkie D Swanson T. Sunitinib and PF-562,271 (FAK/Pyk2 inhibitor) effectively block growth and recovery of human hepatocellular carcinoma in a rat xenograft model. Cancer Biol Ther. (2014) 8:856–65. doi: 10.4161/cbt.8.9.8246, PMID: 19458500

[97] Kanteti R Mirzapoiazova T Riehm JJ Dhanasingh I Mambetsariev B Wang J. Focal adhesion kinase a potential therapeutic target for pancreatic cancer and Malignant pleural mesothelioma. Cancer Biol Ther. (2018) 19:316–27. doi: 10.1080/15384047.2017.1416937, PMID: 29303405 PMC5902231

[98] Li X Ormsby MJ Fallata G Meikle LM Walker D Xu D. PF-431396 hydrate inhibition of kinase phosphorylation during adherent-invasive Escherichia coli infection inhibits intra-macrophage replication and inflammatory cytokine release. Microbiol (Reading). (2023) 169:1–12. doi: 10.1099/mic.0.001337, PMID: 37311220 PMC10333790

[99] Lugones Y Loren P Salazar LA. Cisplatin resistance: genetic and epigenetic factors involved. Biomolecules. (2022) 12:1365–76. doi: 10.3390/biom12101365, PMID: 36291573 PMC9599500

